# Joy, Exercise, Enjoyment, Getting out: A Qualitative Study of Older People's Experience of Cycling in Sydney, Australia

**DOI:** 10.1155/2013/547453

**Published:** 2013-06-20

**Authors:** Alexis Zander, Erin Passmore, Chloe Mason, Chris Rissel

**Affiliations:** ^1^NSW Public Health Officer Training Program, Centre for Epidemiology and Evidence, NSW Ministry of Health, 73 Miller Street, North Sydney, NSW 2055, Australia; ^2^School of Public Health and Community Medicine, UNSW Medicine, The University of New South Wales, Sydney, NSW 2052, Australia; ^3^Council on the Ageing (NSW), P.O. Box A973, Sydney South, NSW 1235, Australia; ^4^Prevention Research Collaboration, School of Public Health, University of Sydney, 92-94 Parramatta Road, Camperdown, NSW 2050, Australia

## Abstract

*Introduction*. Cycling can be an enjoyable way to meet physical activity recommendations and is suitable for older people; however cycling participation by older Australians is low. This qualitative study explored motivators, enablers, and barriers to cycling among older people through an age-targeted cycling promotion program. *Methods*. Seventeen adults who aged 50–75 years participated in a 12-week cycling promotion program which included a cycling skills course, mentor, and resource pack. Semistructured interviews at the beginning and end of the program explored motivators, enablers, and barriers to cycling. *Results*. Fitness and recreation were the primary motivators for cycling. The biggest barrier was fear of cars and traffic, and the cycling skills course was the most important enabler for improving participants' confidence. Reported outcomes from cycling included improved quality of life (better mental health, social benefit, and empowerment) and improved physical health. *Conclusions*. A simple cycling program increased cycling participation among older people. This work confirms the importance of improving confidence in this age group through a skills course, mentors, and maps and highlights additional strategies for promoting cycling, such as ongoing improvement to infrastructure and advertising.

## 1. Introduction

Maintaining the health of Australia's ageing population is a priority for governments and healthcare providers, not only to improve quality of life but also to minimise the burden of ill health on existing resources [1]. Ensuring that older people get the recommended amount of daily physical activity is a key way to improve and maintain health in this important group.

Just 40% of Australian adults who aged 55–74 years achieve the recommended amount of physical activity each week, and after 75 years, the proportion falls to 24% [2]; however identifying physical activities that are enjoyable and accessible for older people can be challenging. Many older adults prefer to exercise alone or with others of their own age [3], and more than half of older Australians undertake their physical activity in unstructured forms such as walking to local shops [4]. Barriers to exercise for older people can include cost, time, and difficulty, travelling to group programs [5].

Cycling is a form of physical activity with particular benefits for older people. It is nonweight bearing and therefore has less impact on the joints than jogging or other running sports [6], and several longitudinal epidemiological studies have shown significant risk reduction for all-cause and cancer mortality, cardiovascular disease, colon and breast cancer, and obesity morbidity in middle-aged and elderly cyclists [7–9]. Cycling may also contribute to improved quality of life for older people [10], by enhancing social networks and building empowerment, and can be incorporated easily into a daily routine [11].

Despite these benefits, cycling participation by older adults in Australia is low [12]. In contrast to many European cities where older people continue to cycle [13], in Australia just 18% of those who aged over 50 cycled in the past year, compared with 50% of those aged 10–29 and 39% of those aged 30–49 years [12].

A small number of published studies have investigated the barriers and facilitators of adult cycling in Australia, but none have looked specifically at the important and growing population of older people. These studies found that poor confidence and cycling ability are concerns for inexperienced riders [14, 15]. Lack of cycle paths is often cited as a barrier to regular cycling, as is fear of cars and negative attitudes from motorists [14, 16, 17].

This study investigated the motivators, barriers and facilitators of cycling in older people who participated in a pilot cycling program. Feedback on the program itself was also sought.

## 2. Methods

This qualitative study was part of a cycling promotion study among older people, looking at improvements in leg strength and balance as risk factors for falls [18]. Adults who aged 50–75 years, who were willing to cycle for two or more hours per week over a 12-week period, were recruited through local advertising and word of mouth in a discrete geographic area of Sydney (Canada Bay), Australia. Canada Bay is an innerwest suburb of Sydney, located around 8 km west of the city. The area scores 1,076.5 on the SEIFA index of disadvantage, indicating that it is less disadvantaged that the national average, and the median/average age of the population is 39 years of age, 2 years above the Australian average [19]. The site was chosen for this study to build on existing relationships between one of the researchers (CM) and the community centre and Bicycle User Group in that area.

We aimed to recruit around 20 participants for the pilot study because it was feasible to support this quantity with the resources available, and the researchers felt that around 20 participants would be sufficient for the purposes of this trial.

### 2.1. Qualitative Methods

Semistructured interviews were conducted one-on-one before participants attended a cycling skills course and 12-weeks later. The study's chief investigator conducted baseline interviews, and follow-up interviews were conducted by another author. The chief investigator was experienced in qualitative interviewing techniques, is personally within the age bracket for participation in the study, and is an experienced cyclist who rides to work daily. The second interviewer has less experience in qualitative interviewing techniques, is younger than the study participants, and cycles to work often.

Five themes were investigated; motivators and barriers were the focus of the baseline interview, while the follow-up interview focused on benefits/outcomes, enablers, and cycling promotion.

Interviews were audio-recorded but not transcribed, and analysed by an individual researcher using thematic analysis. Interviews were coded and then data linked by codes were collated into potential themes. In this way, the researcher generated a thematic map of the entire analysis. Themes were further refined in an ongoing analysis. The person conducting the analysis was younger than the study participants, is female, and cycles to work occasionally.

### 2.2. Program Components

Participants were supported to complete their 2 hr/week of cycling by providing cycling skills training, mentors, and a resource pack.

#### 2.2.1. Cycling Skills Course

The program commenced with a cycling skills course to develop participants' cycling ability, safety knowledge, and confidence, as poor skills and confidence are known barriers to taking up cycling across all ages. The course was delivered by accredited trainers “Bikewise,” and funded by the City of Sydney Council (see http://www.bikewise.com.au/). The course lasted 4.5 hours and covered basic bicycle control skills (starting, braking, and turning), route planning, and on-road riding skills.

#### 2.2.2. Mentors

Participants were matched with mentors from their local area, who were experienced cyclists and could be contacted either directly or via a google group. Each mentor/participant pair was free to meet and/or interact as they chose. The research team also made brief informal phone calls to participants at one and six weeks to maintain participant engagement and identify any additional support required. Strong social support and encouragement have been shown to enable and motivate people to take up and sustain physical activity, and these interventions targeted these constructs.

#### 2.2.3. Resource Pack

Participants received a resource pack which included local cycling maps, information about local Bicycle User Groups, and where and when local group rides were held. These interventions targeted the potential barrier of participants not knowing where to ride and also encouraged participants and supported social connectedness.

## 3. Ethical Approval

This study received ethical approval on September 7, 2011 from the University of Sydney Research Integrity Human Research Ethics Committee (Protocol no.: 09-2011/14065). The approval was ratified by the University of NSW HREC (HREC Ref. # HC12587) on October 25, 2012.

## 4. Results

Seventeen participants were recruited, ranging from 49 to 72 years of age (mean 61 years). The majority (twelve) were female. Most (twelve) had not ridden in the past year, but fifteen were undertaking some form of physical activity; however the amount varied widely, ranging from “a little bit of walking” to one woman running 50 km per week.

Two participants withdrew from the program for personal reasons after the baseline interview, and researchers were unable to schedule a suitable time for a followup interview with four participants. All available interviews were included in this analysis—17 at baseline and 11 at followup.

The four participants who were unable to attend a follow-up interview were at the younger end of the age bracket (more likely to be in their 50s) and more likely to be working.

Nine participants successfully met the 2 hr per week cycling target.

### 4.1. Before the Program

As the sample was self-selected, all enrolled participants were highly motivated to start cycling. The main reason for wanting to cycle was for exercise, and the biggest perceived barrier was fear of cars and traffic.

This study was advertised as a way to potentially improve balance and leg strength, and this should be considered a motivator for people to take part.

#### 4.1.1. Why Cycle? (Motivators)

All participants cited exercise as a major reason to take up cycling, as many did not like gyms and thought that cycling would be a more fun way to exercise, and would fit more easily into their lifestyle.

*“I'm not fantastic on the discipline. If it fits into my lifestyle, then I'll do it and love it ... but if I have to ... go to the gym ... then I'm not very good at following through” (59yo female).*

Some participants added that cycling is a form of exercise that they can continue to do now that they are older.

*“We [my wife and I] used to run ... but I can't run anymore because I damaged one of my knees so I'm past running, so that's why we're thinking well bikes will be good” (62yo male).*

All participants also mentioned enjoyment as a reason to cycle, but this was not as strong a motivator as exercise. Cycling was perceived as a fun way to spend time with family or make new friends. Participants expected to enjoy being outdoors and anticipated the feeling of freedom that cycling brings.

*“I think it's quite nice because I see my husband going off and it's like he has a social group and ... they always seem to stop somewhere nice for coffee ... I think that would be quite nice to be in a similar sort of social group” (67yo female).*

* “Joy, exercise, enjoyment, getting out, you know? Getting out and getting about” (59yo female).*

Around a third intended to use the bicycle for local utility trips. Motivators included convenience, exercise, enjoyment, and the environment.

*“I drive to work, I drive to dad, I drive back from dad, I drive to church, I drive... even to the shops now ... I'm just driving all the time and I'm hating it ... I'd love to ... just jump on my bike to go and get the milk” (59yo female).*

Several participants had plans to go on a cycling holiday with family members and were building their confidence and fitness.

*“I said to my son ... next year, why don't you meet me in Copenhagen and we can cycle up the east coast?” (71yo female).*

#### 4.1.2. Barriers

The primary barrier to cycling for all participants was fear of cars and riding on streets. Most would only consider riding on cycle paths and very quiet roads, and many mentioned friends who had had a “close call” while cycling on roads.

*“I've got a lot of friends ... and I can't find a single friend who thinks that it's safe enough for them to be on the road” (67yo male).*

Many were not confident in their riding ability, mentioning they might be “a bit wobbly” on the bike to begin with because they had not ridden in years and had never ridden a bicycle with gears. Many were relying on the cycling skills course to practice and to build confidence.

*“I feel very nervous about riding a bike because I don't feel I can have adequate control over it ... in terms of wobbling where there's traffic” (67yo female).*

Interestingly, participants did not feel particularly concerned about falling off their bikes as older people. Participants were certainly aware that they may fall, but they reported that their fear was no worse than when they were younger. Even when asked about being frail or having brittle bones, this group were not particularly concerned with this aspect of cycling.

### 4.2. After the Program

Eleven participants completed an interview after the program. Of these, one had been unable to cycle due to an unrelated injury, and one found she lacked the skill to cycle so she gave up on her 2 hours/week target.

All of the remaining nine participants had met their 2 hr per week target and many had done much more. Most were cycling for recreation and fitness, and just over half had also used the bicycle to travel locally to work, the gym, or local shops and events. All who had met their target reported a very positive experience from cycling.

*“you enjoy it, you're getting to work, you're missing the traffic, it makes you feel more healthy in your mind ... and you end up stronger!” (59-year-old female).*

#### 4.2.1. Benefits/Outcomes

Overwhelmingly, taking up cycling was a liberating, fun experience, and many participants reported that it felt lovely to be outdoors, exercising in nature. Many recalled cycling in childhood and recaptured a sense of joy and freedom.

*“I loved the wind blowing through your hair, that was lovely! ... That was the exhilarating part of it” (71-year-old female).*

The exception was the one participant who found she was unable to cycle. This was a distressing realisation for this participant and she expressed disappointment in herself. At baseline, this participant explained that she had fallen from her bike recently and had lost her riding confidence. She hoped that participating in this program would give her the resources and motivation that she needed to get back on the bicycle.

Many participants also enjoyed a benefit to their social life as a result of cycling. They had a new experience to talk about with friends and inspired others to take up cycling themselves. In addition, cycling “plugged participants into” a new community, allowing them to interact with people outside their usual social group. The best example of this was one local cycling group, where two participants rode on weekends with a father and his two daughters. Participants loved interacting with the two young girls, as well as strengthening their own friendship.

Finally, most of the women expressed a strong sense of pride at their cycling achievements and felt empowered by overcoming their fear of cycling and improving their skills, especially as an older person. Friends told them they were an “inspiration,” which heightened the positive association.

*“some of my friends, they're amazed that I've gone out (well, I am too!) ... she said ‘I admire you now for what you've done'” (71yo female).*

Participants were not asked about this feeling specifically; it came up spontaneously in their interviews. Feeling proud and/or empowerment was not mentioned by any of the male participants in their interviews.

Participants reported feeling more energetic, having a better mood, and being able to relax and sleep better after a bike ride. This improvement in mental health was the most commonly reported health-related benefit of cycling; however they also reported improved cardiovascular fitness and feeling stronger, especially in the legs. One individual's diabetic control had improved so much that he was using less insulin.

#### 4.2.2. Enablers

Improving the participants' confidence was key to them for cycling more. Practicing reinforced their skills, and their confidence gradually improved. The strongest determinants of confidence were bike-handling skills and knowing their intended route. The majority began cycling in parks, before moving to cycle paths, and eventually back-roads as confidence increased.

While finding the confidence to cycle was a struggle for some, the cycling skills course was a hugely positive experience for all participants, and for many it was the main factor in getting them “back on a bike”.

*“Fantastic! If it wasn't for that, I wouldn't be cycling now” (67yo female).*

Participants were the happiest when they were grouped with other older riders or riders with a similar level of skill, as they did not feel they were holding the group back and did not feel embarrassed.

*“I did not want to be the duffer in the class” (71yo female).*

Three participants attended the course a second time, and two more asked whether they were allowed to attend again, emphasising the importance of repetition and reinforcement for older riders. They valued the safety knowledge they gained through the course, such as road positioning and how to approach intersections, and practicing basic skills, which improved their confidence. Many had recommended the course to friends and colleagues and could not speak about it more highly.

*“It's probably the best course I've ever done in my life” (66yo male).*

Due to low confidence, participants needed to feel secure about where they were riding. Mentors played a big role by either providing cycle maps or showing the way. For example, one participant's mentor rode with her to work one day, and after that she was able to ride to work a few times a week, always along that same route.

Local cycle maps were also highly valued, but one participant could not use the map given because she could not locate her house on it. Participants were impressed with the well-maintained cycling facilities available in local parks, and some had not known such facilities existed until they saw them on the map.

*“Well, I was amazed [when I saw the map] at the extent of bike paths and cycle ways in Sydney” (71yo female).*

#### 4.2.3. How Can We Encourage Older Adults to Cycle? (Cycling Promotion)

Everyone noted the need for more safe cycle ways, away from traffic.

* “I'm more concerned about traffic than anything, so if anyone in Sydney wants to build more cycleways, I think that's wonderful!” (62yo female).*

Many participants felt that a media campaign about the benefits of cycling would encourage more older people to cycle, but that campaign would need to be backed-up with more cycle ways. Other suggestions included advertising in seniors magazines and at seniors groups and word of mouth or presentations from other older cyclists. They felt the messages needed to be that cycling is achievable, fun, and beneficial for health and that it could be done safely. Despite this, participants noted that many older people would never be responsive to this type of encouragement as cycling is perceived as too dangerous and some older people simply will not undertake physical activity.

## 5. Discussion

The strategies used to promote cycling in this study were simple and inexpensive, mainly linking motivated people to existing resources, but had a high success rate for increasing cycling participation. This work explored motivators, barriers, and outcomes to taking up or increasing cycling and highlighted successful strategies for promoting cycling in older people. Key findings included that cycling promotion for this age group must focus on improving confidence and must consider the need for reinforcement and repetition.

An age-targeted cycling skills course is a very successful strategy. Encouragement for Bicycle User Groups to reach out to older people, widespread availability of cycling maps, and advertising the multiple benefits of cycling are also helpful.

Continued improvement to cycle paths was strongly supported by participants. Fear of cars and traffic is a strong barrier to cycling across all age groups [20] so investment in infrastructure should also have benefits across the population.

The participant who found she was unable to cycle adds weight to the theory that confidence is extremely important in cycling promotion. This participant was given additional time, resources, and encouragement (she was lent a bike to use and strongly encouraged by her mentor); however even with these additional resources she was not able to overcome her fear of falling off her bike again although she persisted in trying. None of the other participants mentioned previous bad experiences with cycling, indicating that a previous bad experience may well cause a significant loss of confidence which would need additional, tailored strategies to overcome.

Another interesting finding is that the sense of empowerment and pride was felt very strongly by female participants but not even mentioned by men. It is possible that men may be inherently more physically confident and assume that they will be able to ride without problems while for women this is a realisation rather than an assumption. It could also be that both sexes felt empowered and proud of cycling but that women were more likely to talk about it at interview, especially as the topic was not specifically discussed or explored.

Contrary to our own preconceived ideas and that of colleagues who we spoke to informally, the participants themselves did not express concerns about being frail and bicycle riding. There may be a feeling among the general population that older people need to be physically cautious that is not held by older people themselves, or worse that older people participating in physical activity are being “reckless”. If this view is held widely, it may partly explain why older people are not routinely targeted and encouraged in physical activity campaigns, despite the many benefits that physical activity provides in older age.

Our study showed that exercise is a strong initial motivator when encouraging older people to cycle, as are sociability and fun, but the best outcomes are empowerment and improved quality of life.

The cycling skills course was an important factor in improving cycling confidence. Participants preferred to be in a group with others like themselves and some needed the reinforcement and repetition of attending the cycling skills course a second time. Following our program, the City of Sydney has set up a cycling skills course specifically for older people, called “Rusty Riders” [21]. Having a mentor for support and encouragement and to ride with a participant along a new route was another effective method to support new riders, and for some new riders, group rides were a fun way to experience the social aspect of riding, gain confidence, and learn about where to ride while getting their physical activity. A public health campaign focusing on the health benefits of cycling and showing personal stories of other older cyclists might be effective and is worthy of further research.

Local cycle maps were highly valued and might be improved if someone (perhaps a mentor) could interpret the map and highlight a route for an older person to take.

As with all qualitative work, our findings are specific to the older people in our study group and not necessarily representative of the broad older population in Sydney. However, the broad themes identified in this group of older adults appear to be readily applicable to older adults generally.

Similarly as with all qualitative work, differences between interviewers may have influenced the way the interviews were conducted and the way participants related to the interviewers.

Interview data were analysed by one researcher, which may be seen as a weakness to the study; however analysis of qualitative data involves interpretation of study findings, and this process is inherently subjective. It is well accepted in qualitative research that a definitive, objective view of social reality does not exist and different researchers may well interpret the same data differently. Therefore validation by an additional researcher would not necessarily give a “correct” interpretation, simply another, equally valid one.

The participants who were lost to followup were all contacted multiple times, but the researchers were unable to schedule a time which was convenient for them for their followup interview. On reflection, these participants were more likely to be younger and working, meaning they were probably busy and had less time to devote to the study. Unfortunately, we are unable to say whether these participants had actually increased their cycling behaviour but an area for further research may be investigating the differences for cycling promotion in working, compared with nonworking older adults.

Other areas for further research could expand on our finding that empowerment, pride, and external feedback (admiration from peers) were very important positive outcomes for female participants but not mentioned by men. Another relevant topic would be looking at the use of electric bikes (ebikes) as enablers (or perhaps deterrents) to cycling in this age group.

## 6. Conclusions

This study suggests that cycling can have a positive influence on the quality of life of older adults, especially through a sense of empowerment and pride, broadening and invigoration of social networks, and simple pleasure. Older people could successfully be encouraged to cycle relatively cheaply and easily by linking them with existing resources in an age-targeted way. While additional cycling infrastructure remains an important goal, in the meantime, more can be done to enable people to use the existing infrastructure. Programs such as the one used here are simple and low-cost to implement. Cycling promotion should centre around improving older people's cycling confidence through improving skills and practice and repetition, promoting existing cycle paths, increasing safe cycle paths, and promoting cycling to older adults for its health, social, and well-being benefits.
